# Iron accentuated reactive oxygen species release by NADPH oxidase in activated microglia contributes to oxidative stress in vitro

**DOI:** 10.1186/s12974-019-1430-7

**Published:** 2019-02-18

**Authors:** Young J. Yauger, Sara Bermudez, Kasey E. Moritz, Ethan Glaser, Bogdan Stoica, Kimberly R. Byrnes

**Affiliations:** 1 Neuroscience Graduate Program, Uniformed Services University of the Health Sciences, Room C2099, 4301 Jones Bridge Road, Bethesda, MD 20814 USA; 2 Department of Anatomy, Physiology, and Genetics, Uniformed Services University of the Health Sciences, Room C2099, 4301 Jones Bridge Road, Bethesda, MD 20814 USA; 3 Department of Anesthesiology and Center for Shock, Trauma, and Anesthesiology Research (STAR), University of Maryland, School of Medicine, 655 W. Baltimore St, Room #6-015, Baltimore, MD USA

**Keywords:** BV2, Iron sulfate, Neuronal toxicity, Microglial polarization, Neuroinflammation

## Abstract

**Background:**

Excessive iron contributes to oxidative stress after central nervous system injury. NADPH oxidase (NOX) enzymes are upregulated in microglia after pro-inflammatory activation and contribute to oxidative stress. The relationship between iron, microglia, NOX, and oxidative stress is currently unclear.

**Methods:**

We evaluated the effects of iron on lipopolysaccharide (LPS)-activated microglia and its secondary effect within neuronal co-cultures. Further, NOX2 and four specific inhibitors were tested to evaluate the relationship with the reactive oxygen species (ROS)-producing enzymes.

**Results:**

An iron dose-dependent increase in ROS production among microglia treated with LPS was identified. Interestingly, despite this increase in ROS, inflammatory polarization alterations were not detected among the microglia after exposure to iron and LPS. Co-culture experimentation between primary neurons and exposed microglia (iron and LPS) significantly reduced neuronal cell number at 24 h, suggesting a profound neurotoxic effect despite the lack of a change in polarization phenotype. NOX2 and NOX4 inhibition significantly reduced ROS production among microglia exposed to iron and LPS and reduced neuronal damage and death in response to microglial co-culture.

**Conclusions:**

In conclusion, iron significantly increased ROS production and neurotoxicity without exacerbating LP-activated microglia phenotype in vitro, suggesting that iron contributes to microglia-related oxidative stress, and this may be a viable therapeutic target for injury or neurodegeneration. Further, this study highlights both NOX2 and NOX4 as potential therapeutic targets in the treatment of iron-induced microglia-related inflammation and neurotoxicity.

**Electronic supplementary material:**

The online version of this article (10.1186/s12974-019-1430-7) contains supplementary material, which is available to authorized users.

## Background

Injury to the central nervous system (CNS), such as a traumatic brain injury (TBI), results in secondary pathophysiology that includes edema, glutamate toxicity, and inflammation. Neuro-inflammation begins within the focal lesion site acutely after injury and can persist chronically [1–4]. A hallmark sign of neuroinflammation is post-injury oxidative stress. Multiple reports of oxidative damage to neurons through carbonylation, nitrosylation, lipid peroxidation, and DNA damage have been observed acutely and chronically in TBI [5–8].

A major contributor to oxidative stress in the CNS are microglia, the parenchymal immune cell of the brain. Microglia can modulate the inflammatory response through cytokines, chemokines, and reactive oxygen species (ROS) [9]. Microglia are known to assume a pro-inflammatory phenotype shortly after injury and can persist for years [2, 10]. Originally described as the respiratory burst, superoxide generation by microglia is an effective modality to mitigate pathogens or other perturbations in the brain [11, 12]. This mechanism is mediated through the activation of the NADPH oxidase 2 (NOX2) enzyme. NOX2 is a member of a family of enzymes consisting of seven isoforms responsible for producing various ROS. Of these family members, two isoforms, NOX2 and NOX4, are present and elevated within microglia after TBI [13]. Although they are isoforms of the same family of enzymes, NOX2 and NOX4 possess significant disparities. NOX2 requires phosphorylation to become activated, followed by multi-subunit assembly before superoxide synthesis can begin. Contrastingly, NOX4 is constitutively active and produces hydrogen peroxide for various cellular activities throughout the cell to maintain homeostasis [14].

Another source of oxidative stress within TBI is iron. Iron is elevated within TBI lesions, particularly among moderate to severe injuries [15, 16]. Under steady state conditions, iron is transported within transferrin and actively transported across the cell membrane where it is subsequently sequestered within ferritin (FER) to protect the cell from its unique physical properties [17]. Excessive extracellular Fe^2+^ not sequestered within proteins is instead transported via divalent metal transporter 1 (DMT1) and delivered to the intracellular compartment [18, 19]. Once in the intracellular compartment, Fe^2+^ is immediately sequestered within FER. If FER reaches its capacity for iron, the remaining unbound iron may undergo a spontaneous non-enzymatic Fenton, Haber-Weiss reaction [20]. Specifically, this reaction requires hydrogen peroxide to oxidize iron to its stable Fe^3+^ form. Following, it can reduce back into Fe^2+^ [21]. More concerning, this non-enzymatic reaction results in the synthesis of hydroxyl radicals, the most reactive form of ROS, which has been implicated as the major destructive ROS within TBI [22–24].

It is well established that iron deposition, oxidative stress, and activated microglia are present within CNS lesions. This triad is considered a major contributor to neural degeneration among many CNS diseases. The exact interactions between the components of the triad are unknown; however, multiple studies purport iron chelation or antioxidant therapies can reduce CNS inflammation leading to improved functional outcomes, reduced lesion volumes, and altered neuronal morphology after injury [25–27]. Since it is generally accepted that improved neuronal survivability following injury may translate into improved long-term outcomes, it is imperative to evaluate the relationship between Fe^2+^, oxidative stress, microglial polarization, and their effect on neuronal survivability. Therefore, we hypothesized that Fe^2+^ can accentuate a ROS-producing pro-inflammatory phenotype in activated microglia, including primary cells or microglial cell lines, in a NOX2- and/or a NOX4-dependent manner, which subsequently reduces neuronal viability in vitro. This experiment involved both primary microglial cells and the BV2 microglial cell line. While the microglial cell line BV2 has been shown to have some marked differences from primary cell line responses [28], it has noted to be an acceptable alternative or adjunct to primary cells in many experimental lines of investigation [29–31], and we herein demonstrate that it responds similarly to primary cells for all outcomes evaluated.

## Methods

### Cell culture

BV2 microglia cell cultures (a gift from Dr. Carol Colton) were maintained with Dulbecco’s modified Eagle medium (Gibco, Gaithersburg, MD), 1% l-glutamine (Gibco), 1% sodium pyruvate (Gibco), 10% fetal bovine serum (FBS) (Hyclone, Logan, UT), and 1% penicillin and streptomycin (Thermo Fisher Scientific, Pittsburgh, PA) [32]. Cells, between passage 5–25, were plated at a density of 1 × 10^5^/ml for all experiments. Before experimentation, the cells rested between 12 and 24 h within an incubator set at 37 °C and a humidified atmosphere augmented with 5% CO_2_.

Primary microglia cultures were isolated from 2-day-old Sprague-Dawley rat pups yielding 90% purity according to methods previously described by our laboratory [33]. All animal protocols received approval from the Uniformed Services University of the Health Sciences (USUHS) Institutional Animal Care and Use Committee (IACUC). Cells were maintained at 37 °C and a humidified atmosphere augmented with 5% CO_2_ in media containing Dulbecco’s modified Eagle medium (Gibco), 1% l-glutamine (Gibco), 1% sodium pyruvate (Gibco), 10% FBS (Hyclone), and 1% penicillin and streptomycin (Thermo Fisher Scientific). Media replacement occurred every 3–5 days.

Primary cortical neurons were prepared according to protocols previously described [34] with a reported purity of approximately 88 ± 4%. Briefly, brains were removed from P18 pregnant Sprague-Dawley rat embryos, then cerebellums and meninges were removed prior to trypsinization. Cells were cultured in neurobasal media (Invitrogen, Carlsbad, CA) supplemented with B-27 (Invitrogen), 0.2% l-glutamic acid monosodium salt hydrate (Sigma), 0.25% glutamine (Invitrogen), and 1% antibiotic-antimycotic Solution Cellgro® (Corning, Manasas, VA). The cells were plated at a density of 5 × 10^5^ cells/ml onto poly-d-lysine (Sigma) treated cell culture plates. All neuronal cultures are fed on day 2 and day 4 with 500 μl of neurobasal media (Invitrogen), 1% antibiotic-antimycotic solution Cellgro® (Corning), and 0.25% glutamine (Invitrogen). Cells were subjected to 24-h co-culture with microglia on day 7 and then harvested for analysis. PC12 neuron-like cells were cultured in DMEM (Gibco) with 5% fetal bovine serum (Hyclone), 5% heat-inactivated horse serum (HIHS, Gibco), and 1% penicillin with streptomycin (Thermo Fisher Scientific). Cells were passaged every 3–4 days between passage 5 and 20. Differentiation protocols were conducted as previously described [35]. On day 7, differentiated cells were subjected to co-culture with microglia.

Co-culture experimentation between microglia and primary neurons or PC12’s was accomplished by plating BV2s into Costar 0.4 μm transwells (Corning Incorporated, Corning, NY) at a density of 1 × 10^5^ cells/ml and treated as described above. After 24 h, BV2 media containing the experimental treatments was replaced with serum-free media and the transwells were transferred to neuronal cultures possessing only 500 μl of media. The BV2’s were co-cultured with the neurons for 24 h and then discarded.

### Treatment

Concentration curves of lipopolysaccharide (LPS), and deferoxamine mesylate (DFO) (Sigma-Aldrich, St. Louis, MO), were conducted to determine the optimal concentration for each chemical (data not shown). Experimental groups were treated with FeSO_4_ tetrahydrate (100 μM, Thermo Fisher Scientific), LPS serotype 026:B6 (100 ng/ml, Sigma-Aldrich), ferrous ammonium sulfate (Fe(NH_4_)_2_(SO_4_)_2_) (100 μM, Fisher Scientific), (sodium sulfate) Na_2_SO_4_ (100 μM, Fisher Scientific), or DFO (250 μM, Sigma-Aldrich). All treatments were added simultaneously and all tests were performed 24 h after treatment. In subsequent experiments, microglia were treated with GKT137831 (50 μM, Genkyotex, Geneva, Switzerland), GSK2795039 (25 μM, GlaxoSmithKline, Brentford, UK), or vehicle dimethyl sulfoxide (DMSO) (≤ 1%, Sigma-Aldrich). These treatments were given simultaneously to the cultures and all assays were conducted 24 h afterwards.

### Reactive oxygen species assays

The ROS assay 2′,7′-dichlorodihydrofluorescein diacetate (H_2_DCFDA, Thermo Fisher) was performed at 10 μM concentration with minor modifications to the manufacturer’s instructions. The cells were incubated for 45 min with the assay components and fluorescence emission was measured on a FLUOstar (BMG LABTECH Inc., Cary, NC) plate reader.

### Cell death assays

Cell culture media was assessed for lactate dehydrogenase (LDH) 24 h after treatment using the Cytotox 96 assay (Promega) according to the manufacturer’s instructions. Absorbance was measured using a ChroMate microplate reader (Awareness Technology Inc., Palm City, FL).

### Cell proliferation count

Cells were detached with Accutase (Innovative Cell Technologies, San Diego, CA) and counted by a TC 20 (Bio-Rad Laboratories, Hercules, CA) automated cell counter.

### Immunocytochemistry

Cells were fixed with 4% paraformaldehyde, and rinsed twice with 1× PBS. Primary antibodies included rabbit CD86 (1 μl/ml; Abcam, Cambridge, MA), rat CD206 (5 μl/ml; Abcam), rabbit Beta III Tublin (5 μl/ml, Abcam), rabbit anti-cleaved caspase-3 (0.5 μl/ml; Abcam), and mouse NeuN (10 μl/ml; Millipore, Billerica, MA). In addition, cell nuclei were stained with DAPI (0.5 μl/ml; Life Technologies, Carlsbad, CA). Fluorescently tagged secondary antibodies (Invitrogen) were visualized with an Olympus BX43 fluorescent microscope with a CellSens Standard imaging program. Five randomly selected images were taken per sample and each image was qualitatively assessed for overall fluorescence. NeuN-positive cells were manually counted using NIH ImageJ (Bethesda, MD).

### Protein analysis

Cells were rinsed, lysed, and proteins were isolated with cell lysis buffer (Cell Signaling Technology; Danvers, MA) containing protease inhibitors (Thermo Scientific). For Western blotting, proteins were separated by gel electrophoresis with polyacrylamide gels (Bio-Rad) and transferred to nitrocellulose membranes (Bio-Rad). Rabbit DMT1 (15 μl/ml; Abcam) was detected with a secondary antibody conjugated with HRP. Resultant bands were captured with the Simple Protein FluroChem E system and analyzed using densitometry (NIH ImageJ) and normalized to a loading control (0.5 μl/5 ml, mouse GAPDH, Millipore).

### Intracellular iron detection

At 6 and 24 h after incubation with FeSO4, intracellular total iron concentration was assessed using the Iron Assay Kit (Abcam), as per the manufacturer’s instructions. Briefly, cells were washed with cold PBS then collected in 700 μl assay buffer and incubated on ice for 5 min. Following, samples were centrifuged to remove debris. Resultant supernatant then was processed with the kit for detection of total iron, detected at 600 nm on a ChroMate microplate reader.

### Cytokine and chemokine analysis

For cytokine analysis, 1000 μg of protein per sample was subjected to a Proteome Profiler Mouse Cytokine Array (R&D Systems, Minneapolis, MN) according to the manufacturer’s instructions. Resultant dots were analyzed using densitometry (NIH ImageJ). All dots were normalized to manufacturer’s standardization regions within the membranes.

### Fluorescence-activated cell sorting

Cells were harvested using Accutase (Innovative Cell Technologies) and stained for viability with FVS510 (0.5ul/ml; BD Biosciences) and the following antibodies conjugated with fluorophores: CD86 with PE (0.5 μl/ml; BD Biosciences), and CD206 with AF647 (1 μl/ml; BD Biosciences). Samples were analyzed with a BD Bioscience fluorescence-activated cell sorting (FACS) cytometer. All data collected were analyzed with FlowJo version 10.1 (FLOWjo, Ashland, OR).

### Comparative RT-PCR

TRIzol® (Invitrogen, Carlsbad, CA) was used to extract mRNA from the cells and mRNA concentrations were measured by a Nanodrop system (Thermo Scientific). A complementary DNA (cDNA) conversion kit (Applied Biosciences, Waltham, MA) used a Veriti thermal cycler (Applied Biosciences) to convert 1 μg of mRNA into cDNA, according to the manufacturer’s suggested protocol. All quantitative real-time PCR (qRT-PCR) data was procured through StepOnePlus Real-Time PCR System (Applied Biosciences), according to the manufacturer’s suggested protocol. All primers were designed by the Primer-Blast tool and synthesized by Integrated DNA Technologies (IDT, Coralville, IA) and listed in Table 1. CD86 measured by Taqman probe (Thermo Fisher) according to manufacturer’s instructions.

**Table 1:** Selected gene sequences for RT-PCR

Gene	Symbol	Forward (5′-3′)	Reverse (5′-3′)
Ferritin, light polypeptide 1	Ftl1	CGGAGGGTCAACATGCTATAA	GGAAGCGAGTACAGTGGGAA
Ferritin, heavy polypeptide 1	Fth1	CAGTGCTTGAACGGAACCC	GTGGTAGTTCTGGCGCACTT
Mannose receptor	Mrc1 (CD206)	GTGGAGTGATGGAACCCCAG	CTGTCCGCCCAGTATCCATC
Arginase 1	Arg1	GTGAAGAACCCACGGTCTGT	GCACCACACTGACTCTTCCA
Chitinase-like 3	Chil3 (YM1)	ATGGAAGTTTGGACCTGCCC	TCCACAGATTCTTCCTCAAAAGC

### Statistics

All experiments were repeated in triplicate unless otherwise stated. All quantitative data are presented as mean ± standard error of the mean. Data were subject to a one-way analysis of variance (ANOVA) with Tukey’s or Dunnett’s multiple comparison’s post-hoc test when appropriate, unless otherwise stated. All statistical tests were performed using the GraphPad Prism Program, Version 6.0 h for Macintosh (GraphPad Software, San Diego, CA). A *p* value < 0.05 was considered statistically significant.

## Results

### Iron independently induces ROS production and accentuates LPS-derived ROS synthesis

**Fig. 1:**
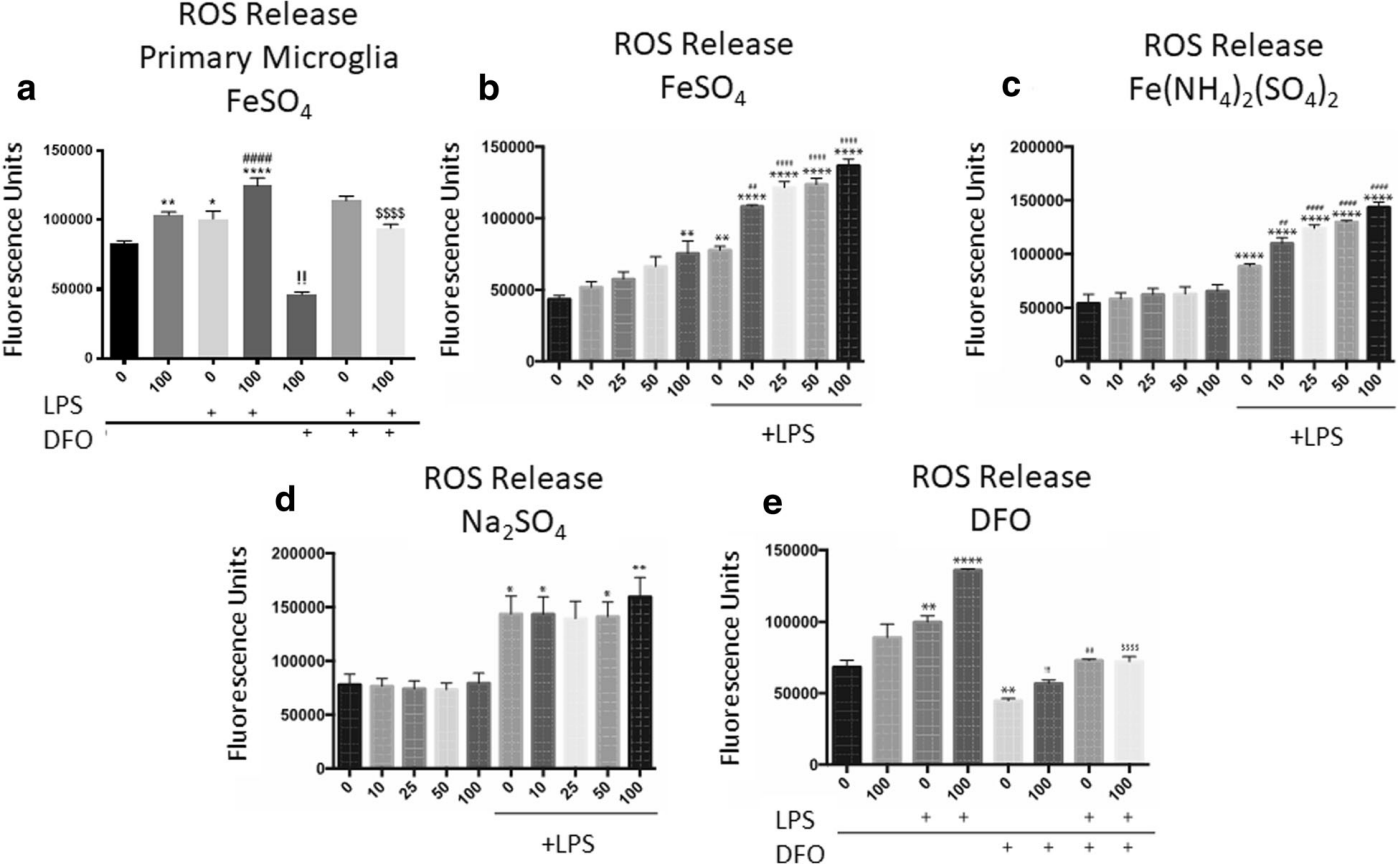
Iron exacerbates ROS generation independently and accentuates LPS-induced ROS production among microglia. **a** Primary microglia show significant elevations in ROS release with FeSO4 exposure. Combining FeSO4 with LPS for 24 h resulted in a compounding effect, with a significant elevation over LPS alone. Treatment with DFO resulted in suppression of the effects of FeSO4, but not in LPS. **b** FeSO_4_ exposure at 100 μM produced a rise in ROS when compared with control (0); LPS also induced an increase in LPS. This increase was elevated further in a concentration dependent manner when microglia were exposed to both FeSO_4_ and LPS. **c** Fe(NH_4_)_2_(SO_4_)_2_ exposure produced similar effects as FeSO_4_. **d** Na_2_SO_4_ did not produce an incremental patterned increase of ROS as previously described. LPS-treated groups did produce an increased amount of ROS, although no differences were noted between the groups treated with LPS. **e** The addition of 250 μM concentrations of DFO reduced ROS concentrations to control levels among all groups. In the graphs, symbols representing significance were assigned according to comparisons: control group (*); LPS group (#); FeSO_4_ (!); and LPS & FeSO_4_ ($). *p < 0.05, **p < 0.01, ***p < 0.001, ****p < 0.0001, ##*p* < 0.01, ####*p* < 0.0001, !!*p* < 0.01, and $$$$*p* < 0.0001. X-axis represents titled drug of graph with μM concentrations. Within the DFO graph the X-axis represents μM concentrations of FeSO_4_. All graphs represent an *n* = 5. All statistics are one-way ANOVA with Tukey post-test. Bars represent mean ± SEM

Microglia are the primary ROS-producing cells in the CNS in response to trauma. Given that they possess the necessary machinery to incorporate iron under basal and LPS-stimulated conditions (Additional file 1: Figure S1), we examined the effect of iron on microglial ROS production. Primary rat microglia cultures were exposed to the Fe^2+^ donor, FeSO_4_, LPS, or both for 24 h. We detected a significant ROS accentuation among the cells with FeSO_4_ exposure that was similar to LPS exposure (Ctrl vs. FeSO_4_, *p* = 0.0027; Ctrl vs. LPS, *p* = 0.0023, one-way ANOVA with Tukey’s post-hoc test, Fig. 1a). Combining FeSO_4_ with LPS for 24 h resulted in a significant elevation of ROS release in comparison to either FeSO_4_ or LPS alone (FeSO_4_ vs FeSO_4_ + LPS, *p* < 0.0001; LPS vs FeSO_4_ + LPS, *p* < 0.0001, one-way ANOVA with Tukey’s post-hoc test, Fig. 1a). Further, administration of the iron chelating agent DFO resulted in significant reduction in ROS production in cells that were exposed to FeSO_4_ (FeSO_4_ vs FeSO_4_ + DFO *p* = 0.0030; FeSO_4_ + LPS vs FeSO_4_ + LPS + DFO *p* < 0.0001, one-way ANOVA with Tukey’s post-hoc test, Fig. 1a).

To determine if the microglial cell line, BV2, responded similarly, BV2 cells were exposed to 0 (control), 10, 25, 50, or 100 μM FeSO_4_ with and without LPS. We found that microglia treated with increasing doses of FeSO_4_ have increased ROS production, reaching significance at a dose of 100 μM. A significant increase in ROS was detected among the groups treated with only 100 μM FeSO_4_ (Ctrl vs 100 μM FeSO_4_, *p* = 0.0047; one-way ANOVA with Tukey’s post-hoc test, Fig. 1b). LPS induced the production of ROS as expected (Ctrl vs. LPS, *p* = 0.0023, Fig. 1b); FeSO_4_ addition to LPS led to an incremental elevation above the LPS-induced ROS in a concentration-dependent fashion (LPS vs: LPS & 10 μM FeSO_4_, *p* = 0.0067; LPS & 25 μM FeSO_4_, *p* < 0.0001; LPS & 50 μM FeSO_4_, *p* < 0.0001; LPS & 100 μM FeSO_4_, *p* < 0.0001; one-way ANOVA with Tukey’s post-hoc test, Fig. 1b). As these initial experiments showed similar results with BV2 cells, we continued experiments utilizing this cell line.

To ensure this phenomenon was not unique to FeSO_4_, another Fe^2+^ donor, ferrous ammonium sulfate (Fe(NH_4_)_2_(SO_4_)_2_), was tested. A similar pattern of increase in ROS within the groups treated with both LPS and Fe(NH_4_)_2_(SO_4_)_2_ was observed (Ctrl vs. LPS, *p* < 0.0001; LPS vs: LPS & 10 μM FeSO_4_, *p* = 0.0033; LPS & 25 μM FeSO_4_, *p* < 0.0001; LPS & 50 μM FeSO_4_, *p* < 0.0001; LPS & 100 μM FeSO_4_, *p* < 0.0001; one-way ANOVA with Tukey’s post-hoc test, Fig. 1c).

Next, to ensure that results were a result of the iron inclusion, a control experiment using inert sodium attached to the sulfate carrier of both iron donors was evaluated by exposing cultures to Na_2_SO_4_. While most groups with LPS treatment demonstrated significant increases in ROS production (Ctrl vs. LPS, *p* = 0.0342; Ctrl vs. LPS & 10 μM Na_2_SO_4_, *p* = 0.0359; Ctrl vs. LPS & 50 μM Na_2_SO_4_, *p* < 0.046; Ctrl vs. LPS & 100 μM Na_2_SO_4_, *p* < 0.0052, one-way ANOVA with Tukey’s post-hoc test, Fig. 1d), there were no differences in Na_2_SO_4_ treated cells.

Finally, to confirm that these effects were the effect of iron, DFO (250 μM), an iron chelator, was assessed. The addition of DFO ameliorated the ROS accentuation by reducing ROS levels among all groups at or below control levels (Ctrl vs. DFO, *p* = 0.0065; FeSO_4_ vs. DFO & FeSO_4_, *p* = 0.0007; LPS vs. DFO & LPS, *p* = 0.0026; FeSO_4_ & LPS vs. DFO & FeSO_4_ & LPS, *p* < 0.0001; one-way ANOVA with Tukey’s post-hoc test, Fig. 1e).

ROS production can be reduced if the numbers of cells are different between experimental conditions. To confirm that iron and/or LPS treatments were not resulting in cell death and subsequent reduction of ROS production, we performed an LDH assay and found no differences in LDH between groups (Additional file 2: Figure S2), indicating that cell death is not impacting ROS production. Increased number of cells can also result in increased ROS production upon stimulation. To verify that the increase in ROS was not due to cell number following stimulation, we counted cells in each group. No significant differences were detected between the groups (data not shown).

### Iron does not induce polarization alterations in LPS-stimulated cells

**Fig. 2:**
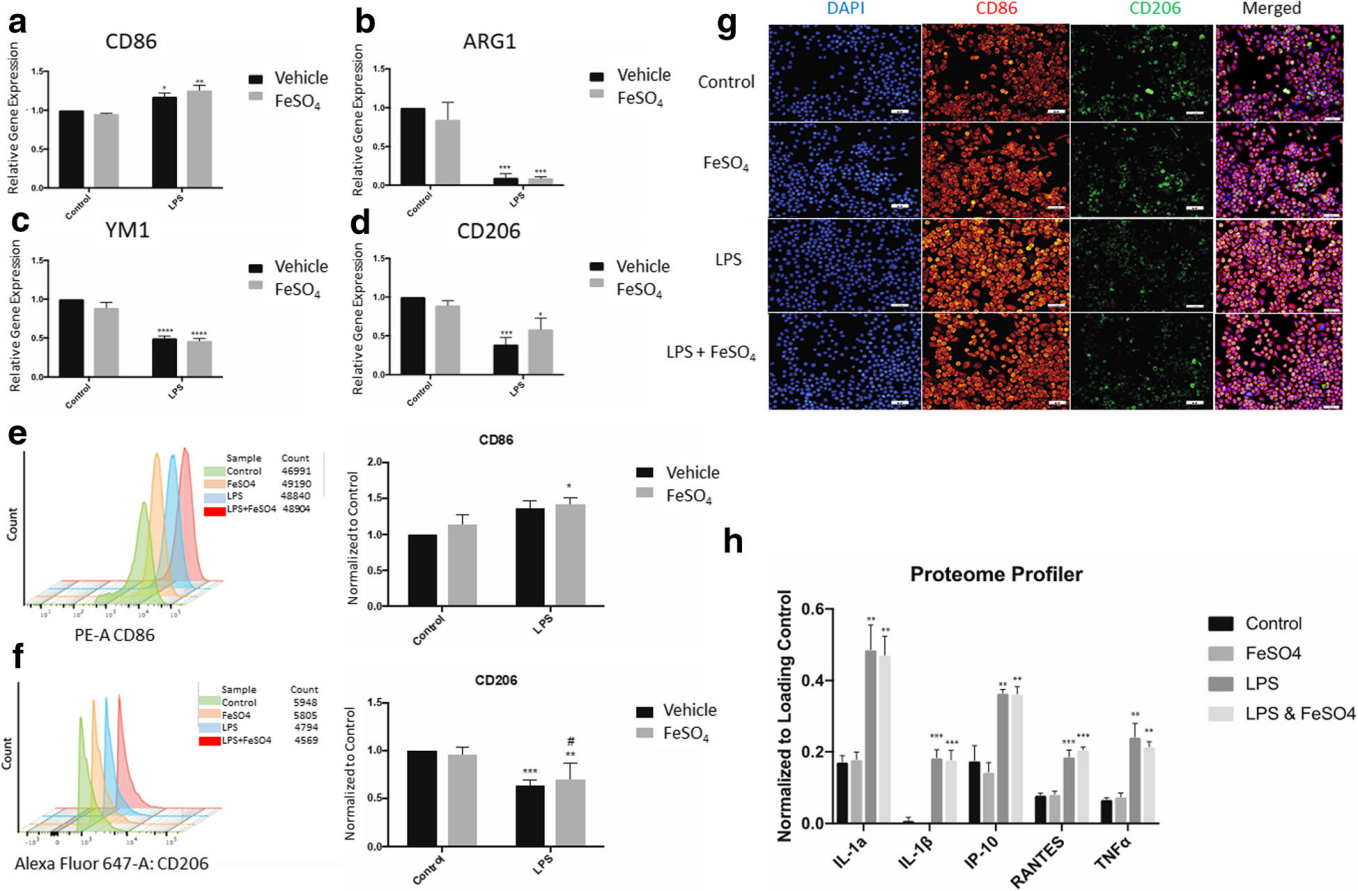
Iron does not alter polarization. LPS promotes a pro-inflammatory gene expression profile, with increased expression of CD86 (**a**) and reduced expression of anti-inflammatory genes ARG1 (**b**), YM1 (**c**), and CD206 (**d**). FeSO_4_ addition had no effect on gene expression. All groups were compared using two-way ANOVA with Tukey post-test. *p < 0.05, **p < 0.01, ***p < 0.001, and ****p < 0.0001. All graphs represent *n* = 5. Flow cytometry showed similar results, wherein LPS did not significantly alter CD86 expression (**e**) alone, but did significantly reduce the expression of anti-inflammatory marker CD206 (**f**). LPS in combination with FeSO_4_ resulted in a significant increase in CD86 expression; there was no combination effect on CD206 expression. Overlays illustrate fluorescence distribution among individual microglia after 24-h exposure to experimental conditions. All groups were compared using two-way ANOVA with Tukey post-hoc test. *p < 0.05; **p < 0.01; ***p < 0.001 represent significant comparisons with Control. #*p* < 0.05 represent significant comparisons with FeSO_4_. *N* = 4 for each marker. Immunocytochemistry demonstrated elevated expression of CD86 among microglia with LPS and reduced CD206 expression (**g**). Iron did not influence CD86 or CD206 protein expression. Representative sample of one trial presented. *N* = 3/group. Size bars represent 50 μm. Finally, Proteome profiler analysis at 24 h after stimulation illustrated a significant pro-inflammatory response to LPS with elevations in IL-1α, IL-1β, IP-10, RANTES, and TNFα (**h**). Data is shown as protein expression normalized to protein loading control. Iron did not affect this response. All groups were compared using two-way ANOVA with Tukey post-test. FeSO_4_ vs. #*p* < 0.05, Control vs. **p < 0.01, ***p < 0.001, *N* = 3. Bars represent mean ± SEM

Microglia rapidly respond to alterations of environmental homeostasis by transitioning to a pro-inflammatory state. This inflammatory state is accompanied by increased cytokine production and has been shown to promote neuronal death and exacerbate the injury. To determine how iron alters microglial polarization in response to LPS, we examined gene expression of several M1 (CD86) and M2 (Arg1, YM1, and CD206) markers 24 h after treatment. LPS resulted in increased gene expression of CD86 and an overall reduction of the anti-inflammatory markers: CD86 (Ctrl vs. LPS, *p* = 0.0402, Fig. 2a), ARG1 (Ctrl vs. LPS, *p* = 0.0002, Fig. 2b), YM1 (Ctrl vs. LPS, *p* < 0.0001, Fig. 2c), and CD206 (Ctrl vs. LPS, *p* = 0.0009, Fig. 2d) (All statistics are two-way ANOVA with Tukey’s post-test). Iron alone did not significantly influence gene expression within any group. In addition, comparisons between LPS versus LPS with iron groups yielded no significant differences, although a slight increase in CD86 was observed with the combination of LPS and iron.

Next, we determined polarization profiles by flow cytometry. CD86 expression in cells treated with LPS alone was slightly elevated over control, although this did not reach statistical significance (Ctrl vs. LPS, *p* = 0.0611, two-way ANOVA with Tukey’s post-hoc test, Fig. 2e). We found the intensity of CD86 staining among groups treated with a combination of LPS and iron increased significantly (Ctrl vs. FeSO_4_ & LPS, *p* = 0.0331, two-way ANOVA with Tukey’s post-hoc test, Fig. 2e). We detected a significant reduction in CD206 fluorescence staining among the groups (Ctrl vs. LPS, *p* < 0.0009; Ctrl vs. FeSO_4_ & LPS, *p* < 0.0047, two-way ANOVA with Tukey’s post-hoc test, Fig. 2f), although no significant effect of iron was found.

To further investigate M1 and M2 marker expression, immunocytochemistry was performed 24 h after incubation with LPS and/or FeSO_4_. Qualitative analysis of the immunocytochemistry revealed a greater expression of CD86 within LPS groups when compared with controls (Fig. 2g). Analysis of CD206 suggested a reduction in its expression when cultures were exposed to LPS. When combined, FeSO_4_ and LPS did not appear to intensify the CD86 fluorescence binding nor did it further reduce CD206 expression.

To confirm these results in primary cells, immunocytochemistry for CD86 was performed. While LPS stimulation did lead to a qualitative increase in CD86 protein expression, addition of FeSO_4_ to LPS-treated primary microglia did not induce a further increase in CD86 (Additional file 3: Figure S3). In fact, qualitative evaluation showed a slight decrease in FeSO_4_-treated cells. No marked difference in cell number was noted in any treatment condition, as assessed by DAPI nuclear staining.

Pro-inflammatory microglia produce cytokines that can be detrimental to neuronal survival. We examined the role of iron on cytokine production in the absence and presence of iron and/or LPS. Six cytokines associated with the pro-inflammatory response were increased by LPS (Table 2). Overall, LPS had a pro-inflammatory effect on microglia—inducing synthesis of IL-1α (Ctrl vs. LPS, *p* = 0.0055), IL-1β (Ctrl vs. LPS, *p* = 0.006), IP-10 (Ctrl vs. LPS, *p* = 0.0053), RANTES (Ctrl vs. LPS, *p* = 0.0006), and TNFα (Ctrl vs. LPS, *p* = 0.0017, two-way ANOVA with Tukey’s post-hoc test, Fig. 2h). Iron alone did not have an effect on any of the cytokines tested, further supporting the idea that iron’s effects are primarily related to ROS release.

**Table 2:** Cytokine and chemokine responses

Protein	Control Mean ± SEM	FeSO_4_ Mean ± SEM	LPS Mean ± SEM	LPS & FeSO_4_ Mean ± SEM
G-CSF	ND	ND	0.074 ± 0.013	0.067 ± 0.008
sICAM-1	0.690 ± 0.254	0.813 ± 0.32	0.813 ± 0.324	0.698 ± 0.26
IL-1α	0.171 ± 0.018	0.179 ± 0.021	0.486 ± 0.069	0.47 ± 0.053
IL-1β	ND	ND	0.183 ± 0.023	0.177 ± 0.027
IL-1ra	0.934 ± 0.052	0.87 ± 0.059	1.165 ± 0.023	1.18 ± 0.104
IP-10	0.175 ± 0.042	0.143 ± 0.026	0.365 ± 0.011	0.362 ± 0.021
M-CSF	0.097 ± 0.01	0.107 ± 0.005	0.085 ± 0.004	0.083 ± 0.011
JE	0.573 ± 0.05	0.535 ± 0.055	0.619 ± 0.049	0.568 ± 0.062
MIP-1α	0.941 ± 0.016	1.07 ± 0.115	0.849 ± 0.064	0.769 ± 0.019
MIP-1β	0.023 ± 0.011	0.021 ± 0.012	0.113 ± 0.028	0.106 ± 0.024
MIP-2	ND	ND	0.072 ± 0.01	0.072 ± 0.002
RANTES	0.079 ± 0.005	0.081 ± 0.009	0.186 ± 0.019	0.206 ± 0.008
SDF-1	0.039 ± 0.008	0.038 ± 0.007	0.04 ± 0.009	0.036 ± 0.006
TIMP-1	0.052 ± 0.0007	0.057 ± 0.01	0.058 ± 0.0029	0.063 ± 0.003
TNFα	0.067 ± 0.005	0.074 ± 0.011	0.241 ± 0.038	0.214 ± 0.014
TREM-1	0.08 ± 0.002	0.08 ± 0.007	0.103 ± 0.003	0.091 ± 0.008

Values represent relative expression of cytokines and chemokines normalized to manufacturer’s controls after stimulation. No significant differences were noted between control and FeSO_4_ or between LPS and FeSO4 & LPS groups. All groups were compared using two-way ANOVA with Tukey’s post-test. All groups represent *N* = 3. All data presented as mean ± SEM

### Iron increases neurotoxicity among co-cultures

**Fig. 3:**
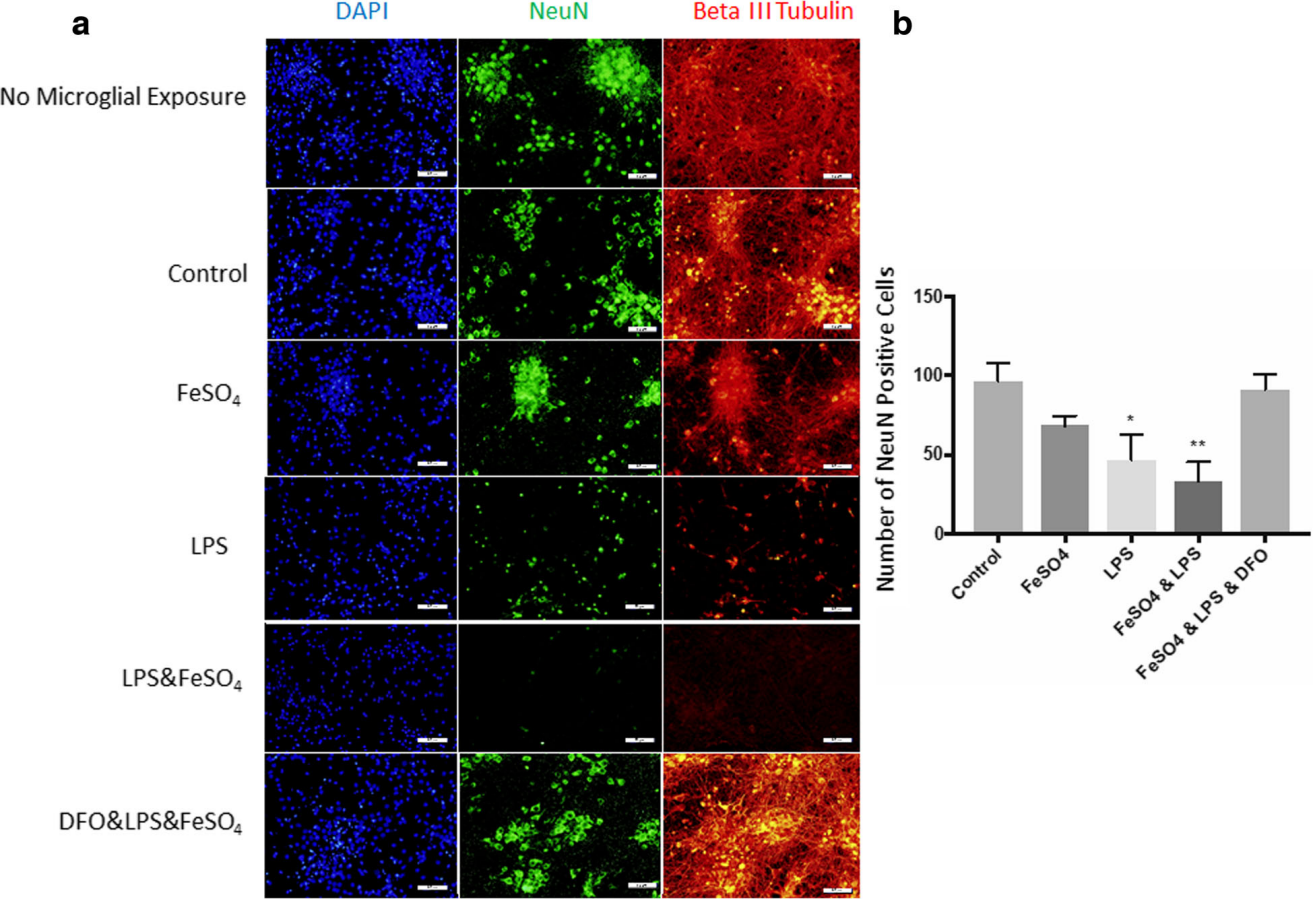
Microglia treated with LPS & FeSO_4_ increased primary neuronal toxicity in co-culture. **a** Immunocytochemistry of NeuN-positive primary neurons after 24-h microglial co-culture revealed reduced number of neurons and neurite network when exposed to LPS or LPS & FeSO_4_. Although FeSO_4_ alone did not result in a significant reduction, a lower number of neurons and decreased density of neurite network was noted. The addition of DFO ameliorated the toxic effect of microglia treated with LPS alone and LPS & FeSO_4_. **b** Quantification of NeuN-positive cells illustrated the toxic effect of LPS alone and in combination with FeSO_4_. Representative sample of 1 trial of 4 shown. Graph represents a sample size of 4. *p < 0.05; **p < 0.01, one-way ANOVA with Tukey’s post-hoc test. Bars represent mean ± SEM. Size bars represent 50 μm

Given that activated microglia promote oxidative stress-induced cell death in neurons, we wanted to evaluate the role of iron on microglia-induced neuronal cell death. We found that when LPS-treated microglia were indirectly co-cultured with primary neuronal cells via transwell plates, the number of NeuN^+^ cells were decreased. Immunocytochemistry for NeuN-positive cells revealed that the addition of iron to the LPS stimulated microglia further reduced NeuN staining, with an almost complete loss of NeuN staining (Fig. 3; no microglia (NM) vs. FeSO_4_ & LPS, *p* = 0.0035; Ctrl vs. FeSO4 & LPS, *p* = 0.0027). This effect was reversed by the administration of the iron chelator DFO to the stimulated microglia, prior to incubation with neurons (LPS & FeSO_4_ vs. DFO & LPS & FeSO_4_, *p* = 0.0064, Fig. 3).

Beta III tubulin is a protein found in neurons that allow visualization of the neurite networks. Staining of primary neurons following co-culture with microglia treated with iron resulted in a marked loss of Beta III tubulin staining. LPS incubation with microglia prior to co-culture further reduced staining, and staining was almost completely lost with the combination of iron and LPS. This observation was again completely reversed with administration of DFO to microglia (Fig. 3a).

### NOX2 or NOX4 inhibition reduces ROS production among microglia exposed to FeSO_4_ and LPS

**Fig. 4:**
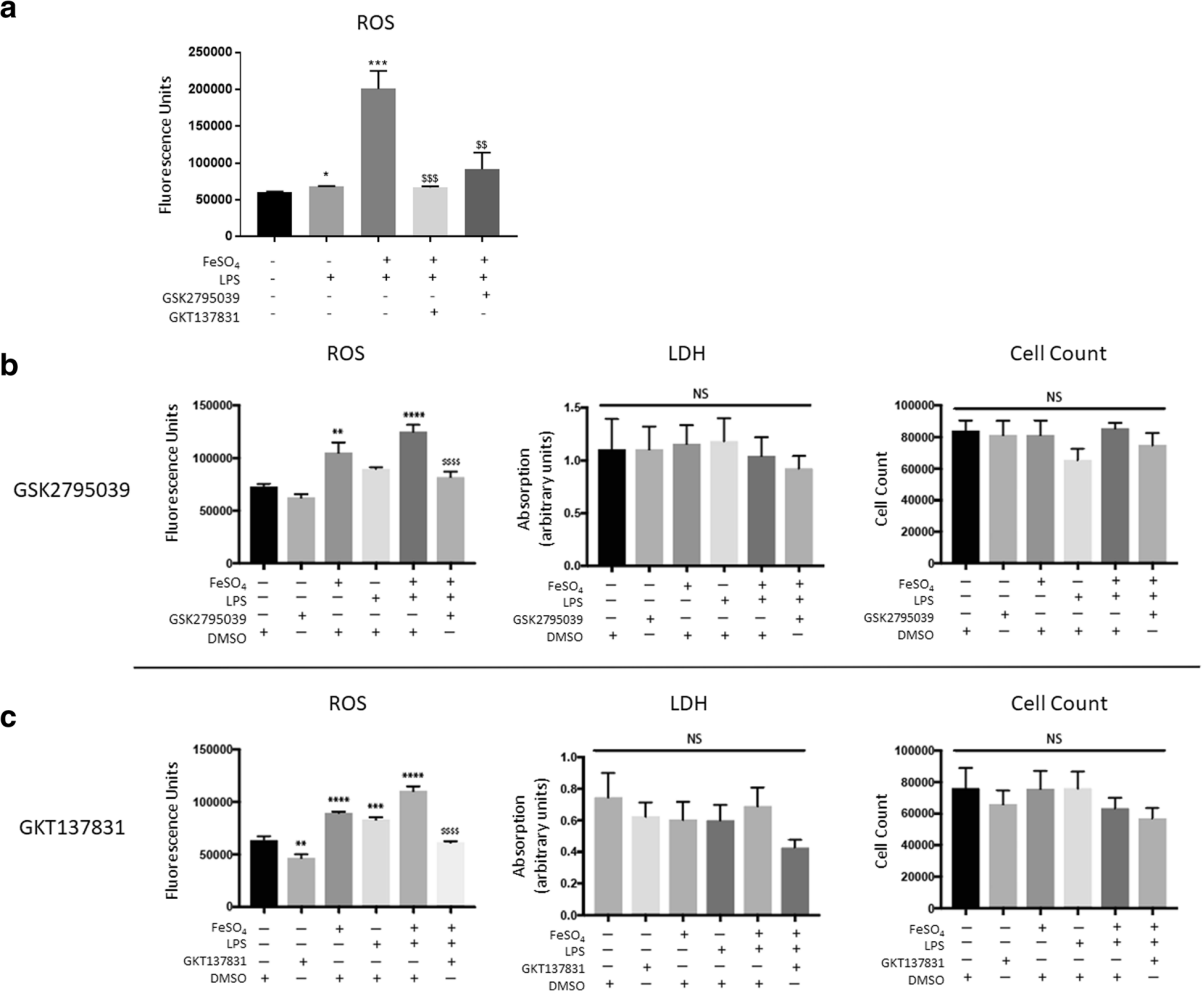
Iron accentuated ROS generation is significantly attenuated by NOX2 and NOX4 inhibition among activated microglia. **a** FeSO_4_ treatment in primary microglia led to a combinatorial effect with LPS that was diminished completely by GSK2795039 and GKT137831. **b** FeSO_4_ treatment among microglia induced a significant increase in ROS. LPS & DMSO did not increase ROS levels alone; however, the addition of FeSO_4_ to the group significantly increased ROS production. NOX2 inhibition with GSK2795039 significantly reduced ROS production to baseline. No significant differences in LDH or cell counts were detected between treatment groups within the GSK2795039 experiments. **c** Robust ROS production among microglia treated with FeSO_4_, LPS alone, or in combination. The addition of NOX4 inhibition of GKT137831 significantly reduced the presence of ROS. No significant differences in LDH or cell counts were detected between treatment groups within the GKT137831 experiments. In all graphs, symbols representing significance were assigned according to comparisons: DMSO group (*); FeSO_4_ & DMSO (#); and FeSO_4_ & LPS & DMSO ($). **p < 0.01, ***p < 0.001, ****p < 0.0001, ^###^*p* < 0.001, and ^$$$$^*p* < 0.0001. All groups were compared using one-way ANOVA with Tukey’s post-hoc test. All graphs represent *n* = 6. Bars represent mean ± SEM

NOX2 is an inducible superoxide generating enzyme that produces hydroxyl radicals and ROS; its structure suggests a partial dependence on iron [36]. Given that NOX2 is upregulated following LPS treatment and iron can accentuate LPS-induced ROS production (Additional file 1: Figure S1a), we treated primary microglia with a NOX2 inhibitor, GSK279503, and measured ROS production. FeSO_4_ and LPS resulted in a significant increase in the presence of ROS (*p* = 0.0002, one-way ANOVA with Sidak’s multiple comparisons post-hoc test, Fig. 4a). NOX2 inhibition of LPS/iron-treated microglia resulted in ROS levels that returned to those of the control group (FeSO_4_ & LPS vs. FeSO_4_ & LPS & GSK, *p* = 0.0015, one-way ANOVA with Sidak’s multiple comparisons post-hoc test, Fig. 4a).

In BV2 cells, FeSO_4_ and/or LPS also resulted in a significant increase in the presence of ROS (DMSO vs: FeSO4, *p* = 0.0089; FeSO4 & LPS, *p* < 0.0001, one-way ANOVA with Tukey’s post-hoc test, Fig. 4b). NOX2 inhibition of LPS/iron-treated microglia resulted in ROS levels that returned to those of the control group (FeSO_4_ & LPS vs. FeSO_4_ & LPS & GSK, *p* < 0.0001, one-way ANOVA with Tukey’s post-hoc test, Fig. 4b). No significant differences were noted between groups by LDH assay or cell counts among cultures (Fig. 4b).

NOX4, unlike NOX2, is constitutively expressed and catalyzes the formation of hydrogen peroxide [36]. Given that hydrogen peroxide can oxidize iron to its reactive state, we sought to determine if NOX4 inhibition could block iron-accentuated ROS production in microglia. We found that NOX4 inhibition with GKT137831 significantly reduced the iron-induced ROS production in primary cells (FeSO_4_ & LPS vs. FeSO_4_ & LPS & GSK, *p* = 0.0003, one-way ANOVA with Sidak’s multiple comparisons post-hoc test, Fig. 4a).

In BV2 cells, we found a similar result. FeSO_4_ and/or LPS led to a significant increase in ROS (DMSO vs: FeSO_4_, *p* < 0.0001; LPS, *p* = 0.001; FeSO_4_ & LPS, *p* < 0.0001, one-way ANOVA with Tukey’s post-hoc test, Fig. 4c). Interestingly, GKT137831 treatment alone resulted in a significant reduction in ROS in comparison to the vehicle-treated group (DMSO vs. GKT, *p* = 0.0055, one-way ANOVA with Tukey’s post-hoc test, Fig. 4c), likely because NOX4 is endogenously active in microglia. In addition, GKT137831 significantly reduced ROS production among microglia simultaneously treated with FeSO_4_ and LPS (FeSO_4_ & LPS & DMSO vs. FeSO_4_ & LPS & GKT, *p* < 0.0001, one-way ANOVA with Tukey’s post-hoc test, Fig. 4c). No significant differences were noted between groups by LDH assay or cell counts among cultures within the GKT137831 set of experiments (Fig. 4c). While slight differences in ROS responses were noted between experiments represented in Fig. 4b, 4c, likely due to slight variability in the control group as a result of passage number, overall trends were similar between the two studies.

### Iron accentuated, microglial-mediated neuronal toxicity is reversed by NOX2 inhibition

**Fig. 5:**
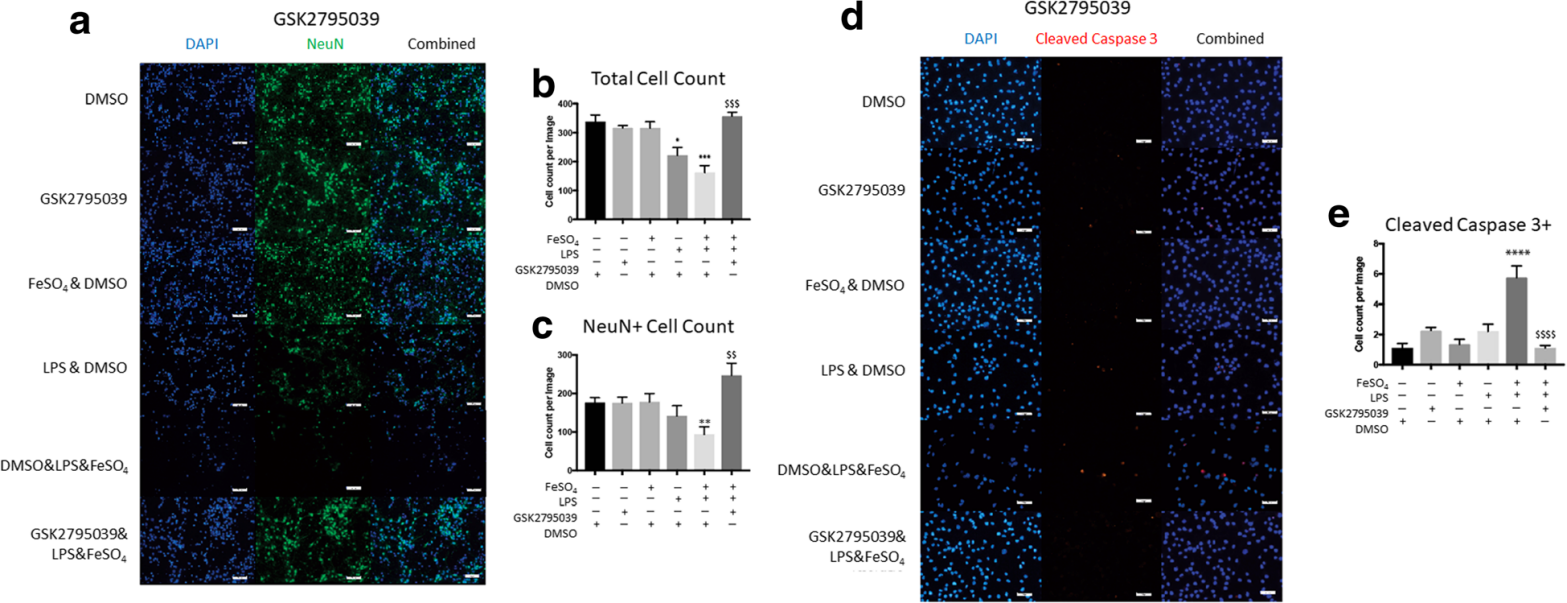
GSK2795039 improves neuronal survival in co-culture with activated microglia. **a** Primary neurons were co-cultured with BV2 microglia exposed to FeSO4, DMSO, LPS, and/or GSK2795039. Immunocytochemistry was completed for neurons (NeuN) at 24 h after co-culture. All groups were counter-stained with DAPI. Qualitatively, a reduction in the expression of NeuN was noted on those groups co-cultured with microglia treated with FeSO4, LPS, or LPS&FeSO4. The addition of GSK2795039 significantly improved neuronal survival. **b** Improved survival among all cell types present in the neuronal culture (DAPI-positive) was observed as increased cell counts in groups treated with GSK2795039. **c** Quantitative analysis of NeuN-positive cell counts revealed a significant reduction in neuronal cell count after co-culture with microglia treated with FeSO_4_ & LPS & DMSO. GSK2795039 prevented neuronal loss after co-culture and significantly improved neuronal cell counts. **d** PC12 cells were differentiated to a neuronal like phenotype for one week prior to microglia co-culture. Immunocytochemistry revealed increased staining for cleaved caspase-3 among PC12 co-cultured with BV2 microglia treated with FeSO_4_, LPS, or LPS&FeSO4. The addition of GSK2795039 to the microglia treatment groups reduced the presence of cells positive for cleaved caspase-3. **e** Quantitative analysis of cleaved caspase-3 positive PC12 cells showed significant increase when co-cultured with microglia treated with FeSO_4_ & LPS & DMSO. However, this increased apoptosis among PC12 cells was reduced by the addition of GSK2795039. All immunocytochemistry images are representative of one trial. All groups were compared using one-way ANOVA with Tukey’s post-hoc test. In all graphs, symbols representing significance were assigned according to comparisons: DMSO group (*) and FeSO_4_ & LPS & DMSO ($). *p < 0.05, **p < 0.01, ***p < 0.001, ****p < 0.0001, ^$$^*p*< 0.01, ^$$$^*p*< 0.001, ^$$$$^*p*< 0.0001. All graphs represent *n* = 4. Bars represent mean ± SEM. Size bars represent 50 μm

To determine the effectiveness of specific NOX isoform inhibition in microglia on neuronal survival, we indirectly co-cultured microglia in transwell plates with primary rat neuron cultures. Microglia pretreated with FeSO_4_ & LPS prior to co-culture with neurons resulted in a reduced nuclear staining of DAPI and neuron specific NeuN (Fig. 5a). DAPI-positive cells were quantified and a significant reduction of cells was noted among neuronal cultures co-cultured with microglia treated with FeSO_4_ & LPS (DMSO vs. FeSO_4_ & LPS & DMSO, *p* = 0.0007; one-way ANOVA with Tukey’s post-hoc test, Fig. 5b). NOX2 inhibition by GSK2795039 within microglia prior to co-culture significantly increased the presence of cells within the neuronal cultures (FeSO_4_ & LPS & DMSO vs. GSK2795039 & FeSO_4_ & LPS, *p* = 0.0012, one-way ANOVA with Tukey’s post-hoc test, Fig. 5b). To determine the toxicity specific to neurons, all NeuN-positive cells were counted among the cultures. Significant reductions of primary neuronal cell counts were detected among cultures treated with a combination of FeSO_4_ & LPS (DMSO vs. FeSO_4_ & LPS & DMSO, *p* = 0.0027, one-way ANOVA with Tukey’s post-hoc test, Fig. 5c). Interestingly, the addition of GSK2795039 significantly increased the presence of neurons among those treated with FeSO_4_ & LPS (FeSO_4_ & LPS & DMSO vs. GSK2795039 & FeSO_4_ & LPS, *p* < 0.0001, one-way ANOVA with Tukey’s post-hoc test, Fig. 5c).

Due to the heterogeneous cellular composition of primary neuronal cultures, we utilized robust differentiated PC12 neuronal-like cells in co-culture with microglia to evaluate the severity of cellular toxicity among purified neuronal cell cultures. To evaluate changes in apoptosis following treatment, we analyzed levels of cleaved caspase-3, a known marker of apoptosis. After co-culture for 24 h, PC12s did not display significantly increased levels of cleaved caspase-3-positive cells when co-cultured with microglia previously treated with vehicle, FeSO_4_, LPS, or GSK2795039 alone. However, a combination FeSO_4_ & LPS treatment of microglia prior to co-culture increased the number of cleaved caspase-3-positive cells within the PC12 cultures; the addition of GSK2795039 reversed this effect (Fig. 5d, e). Caspase-3 gets cleaved and translocated to the nucleus after the induction of apoptosis, so to quantify the differences in apoptosis between treatment groups, all co-localized DAPI and caspase-3-positive cells were counted and the combinatory effect of FeSO_4_ and LPS resulted in significantly increased levels of apoptosis (DMSO vs FeSO4 & LPS & DMSO, *p* < 0.0001, one-way ANOVA with Tukey’s post-hoc test, Fig. 5e). The addition of the NOX2 inhibitor GSK2795039 significantly reduced the presence of caspase-3-positive PC12 cells (FeSO4 & LPS & DMSO vs. GSK2795039 & FeSO4 & LPS, *p* < 0.0001, one-way ANOVA with Tukey’s post-hoc test, Fig. 5e).

### Iron accentuated, microglial-mediated neuronal toxicity is reversed by NOX4 inhibition

**Fig. 6:**
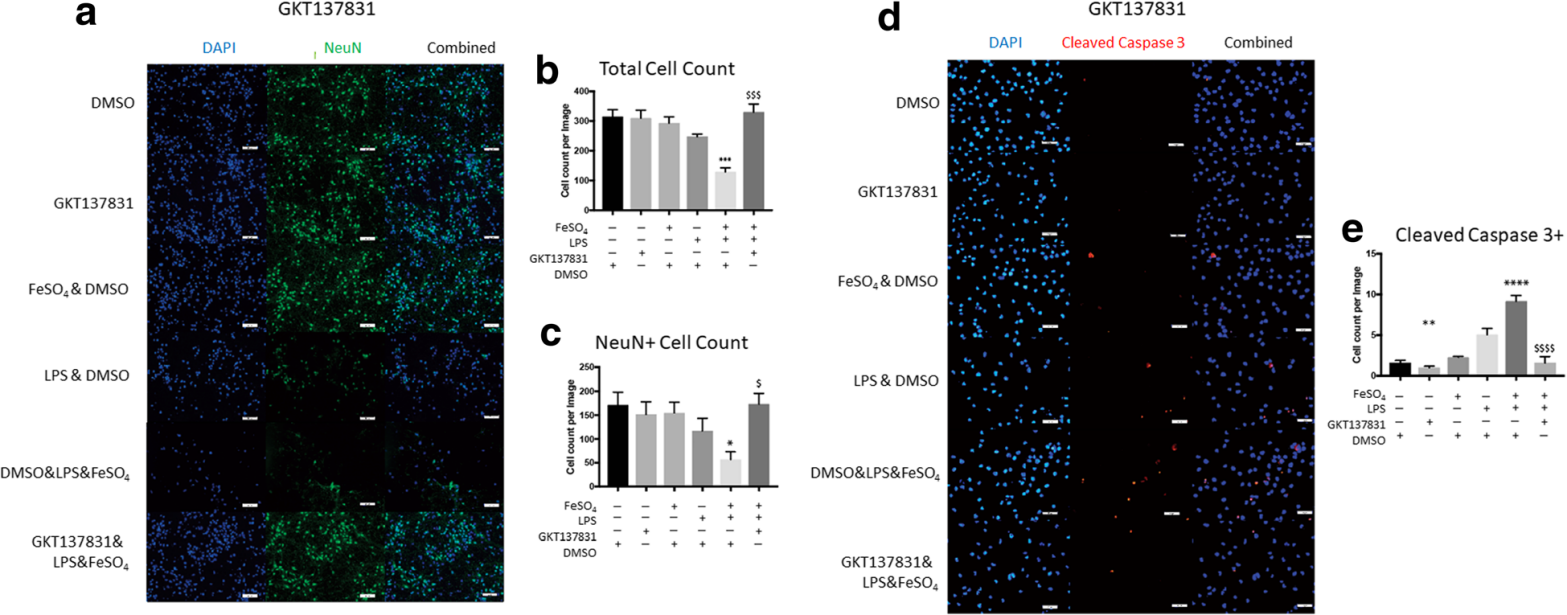
GKT137831 improves neuronal survivability in co-culture with activated microglia. **a** Primary neurons were co-cultured with microglia exposed to FeSO4, DMSO, LPS, GKT, or in combination. Immunostaining of neurons after 24 h co-culture showed a reduction in the expression of NeuN in groups co-cultured with microglia treated with FeSO_4_ & LPS & DMSO. **b** Improved survivability among all cell types present in the neuronal culture were observed by the increased cell counts in groups treated with GKT137831. **c** Quantitative analysis of NeuN counts revealed a significant reduction in the presence of neurons after co-culture with microglia treated with FeSO_4_ & LPS & DMSO. GKT137831 reduced neuronal toxicity and prevented neuronal loss after co-culture. **d** PC12 cells were differentiated to a neuronal like phenotype for one week prior to microglia co-culture. Immunocytochemistry revealed increased staining among PC12 co-cultured with microglia treated with LPS, or in combination with FeSO_4_. The addition of GKT137831 to the microglia treatment groups reduced the presence of cells positive for cleaved caspase-3. **e** Quantitative analysis of PC12 cells positive with cleaved caspase-3 were significantly increased when co-cultured with microglia treated with FeSO_4_ & LPS & DMSO. However, this increased apoptosis among PC12 cells was reversed by the addition of GKT137831. All immunostaining images are representative of one trial. All groups were compared using one-way ANOVA with Tukey post-test. In all graphs, symbols representing significance were assigned according to comparisons: DMSO group (*); FeSO_4_ & DMSO (#); LPS & DMSO (!); and FeSO_4_ & LPS & DMSO ($). **p < 0.01, ****p < 0.0001, ^####^*p* < 0.0001,^!!^*p* < 0.01, and ^$$$$^*p* < 0.0001. All graphs represent *n* = 3. Bars represent mean ± SEM. Size bars represent 50 μm

Next, we evaluated the contribution of NOX4 to microglial-mediated neurotoxicity by inhibiting NOX4 with GKT137831. Primary neuronal cultures displayed fewer DAPI-positive and NeuN-positive nuclei after co-culture with microglia treated with LPS or FeSO_4_ & LPS. This toxic phenomenon was completely reversed by the addition of GKT137831 (Fig. 6a). The DAPI-positive cells were again counted and a significant reduction in cells were noted among neuron cultures exposed to microglia treated with a combination of FeSO_4_ & LPS (DMSO vs. FeSO_4_ & LPS & DMSO, *p* = 0.0005, one-way ANOVA with Tukey’s post-hoc test, Fig. 6b). While cultures were treated identically as in the NOX2 inhibition experiment, slight variation in cell responses resulted in a lack of significant reduction in DAPI-positive and NeuN-positive cells with LPS treatment alone. However, a similar trend was observed. The addition of GKT137831 resulted in a significant increase in the presence of cells (FeSO_4_ & LPS & DMSO vs. GKT137831 & FeSO_4_ & LPS, *p* = 0.0002, one-way ANOVA with Tukey’s post-hoc test, Fig. 6b).

NeuN-positive primary neurons were also counted to determine cellular toxicity among neurons. Those neuronal cultures subjected to co-culture with microglia pretreated with FeSO_4_ & LPS showed a significant reduction in neuronal cell counts which was ameliorated by the addition of GKT137831 (DMSO vs. FeSO_4_ & LPS & DMSO, *p* = 0.048; FeSO_4_ & LPS & DMSO vs. GKT137831 & FeSO_4_ & LPS, *p* = 0.0438, one-way ANOVA with Tukey’s post-hoc test, Fig. 6c).

PC12 cultures were then subjected to NOX4 inhibition by GKT137831. PC12s qualitatively did not display increased levels of caspase-3-positive cells and were not affected by co-cultured microglia previously treated with vehicle, FeSO_4_, or GKT137831 alone. LPS and FeSO_4_ & LPS groups did present with higher numbers of caspase-3-positive cells, which was reversed with the addition of GKT137831 (Fig. 6d). Quantitative analysis demonstrated that LPS treated microglia caused a significant increase in caspase-3-positive PC12 cells (DMSO vs. LPS & DMSO, *p* = 0.0081, one-way ANOVA with Tukey’s post-hoc test, Fig. 6e). This toxicity worsened with the combination FeSO_4_ and LPS treatment groups (DMSO vs. FeSO4 & LPS & DMSO, *p* < 0.0001, one-way ANOVA with Tukey’s post-hoc test, Fig. 6e) and was reversed with the addition of GKT137831 (FeSO4 & LPS & DMSO vs. GKT137831 & FeSO4 & LPS, *p* < 0.0001, one-way ANOVA with Tukey’s post-hoc test, Fig. 6e). In comparison to the NOX2 inhibition experiment (Fig. 5), experimental variability did result in a greater number of cleaved caspase-3-positive PC12 cells in the current experiment, but trends of responses remain the same. This difference may be due to the slight difference in PC12 passage number between NOX2 and NOX4 experimentation.

## Discussion

These data demonstrate that iron contributes to microglial ROS production in a NOX2- and NOX4-dependent manner and that this ROS production, without an accompanying alteration in pro-inflammatory/M1 polarization markers or cytokines, contributes to microglial-induced neurotoxicity. Further, while iron both induces ROS production from microglia and accentuates ROS synthesis from activated microglia, iron did not alter polarization in non-stimulated or LPS stimulated primary microglia or BV2 cells. Importantly, despite this lack of polarization change, the data from the current work demonstrate that the elevated ROS leads to increased neuronal death in a BV2-primary neuron co-culture model and can be reversed with iron chelation. NOX2 and NOX4 may be important within the mechanism of iron accentuated ROS production and their inhibition seems to be effective in reducing neuronal damage.

Iron overload prior to CNS injury exacerbates lesion volume and reduces functional recovery, and suggests a potential priming effect by iron [10]. Our data is consistent with these observations in that the presence of iron exacerbated the ROS production among microglia and exacerbated neuronal death, which may lead to increased lesion volume and reduced functional recovery. This iron accentuated ROS production among microglia exposed to FeSO_4_ & LPS & DMSO was significantly reduced following treatment with GSK2795039, suggesting that the presence of superoxide is essential for the mechanism in which iron can accentuate ROS production. NOX4 inhibition was equally effective in reducing iron accentuated ROS production, suggesting that hydrogen peroxide produced by NOX4 contributes to the mechanism of iron accentuated ROS production as well. Overall, the effective reduction of ROS after GSK2795039 and GKT137831 supports our hypothesis that iron undergoes the Fenton, Haber-Weiss reaction utilizing NOX2- and NOX4-derived superoxides and hydrogen peroxide to accentuate ROS production through positive feedback with NOX proteins. It is also important to note that GKT137831 has been described as dual NOX1 and NOX4 inhibitor [37]. However, NOX1 gene expression was not detected in quantitative polymerase chain reaction among BV2 microglia (data not shown), which suggests that NOX1 inhibition is not likely the result of GKT137831 administration in microglia.

The anti-inflammatory effect of iron chelation suggests that iron contributes to a pro-inflammatory response in CNS injury. However, little is known of iron’s effect on polarization of microglia. To determine iron’s contribution to polarization alterations among microglia, we collected gene expression, FACS, immunocytochemistry, and cytokine/chemokine data. Interestingly, iron alone or in combination with LPS did not significantly alter the gene or protein expression of any polarization or cytokine/chemokine markers assessed in either BV2 or primary microglial cells. This may be due to the overwhelming response to LPS by the cells and therefore a ceiling effect is created that additional stimulation by iron cannot surpass or that iron and LPS do not have an additive effect for these outcomes. The observation of a slight decrease in CD86 staining in primary microglia was interesting and suggests that the ceiling effect is not the true cause, however. Further experimentation into the role of iron in microglial cells is warranted.

Despite the lack of an exacerbated polarization response among microglia, an increased neurotoxic effect was observed among neurons exposed to microglia treated with a combination of LPS & FeSO_4_. This is likely due to the increased oxidative stress in neurons as a result of increased microglial ROS synthesis. Oxidative stress has been identified as a major contributor to neuronal demise after intracerebral hemorrhagic injury [38]. In 2005, Nakamura et al. reported increased DNA damage after ferrous iron infusion into basal ganglia, which was ameliorated by iron chelation therapy [39]. In addition, this increased oxidative stress phenomena is similar in Parkinsonian neurodegeneration where chronically activated microglia are simultaneously present with increased concentrations of iron [40–42]. This may possibly explain why iron exacerbates CNS injuries and disease.

To further support this conclusion, the observed amelioration of neuronal death by the addition of DFO illustrates the importance of iron mitigation following injury [42, 43]. Multiple studies have found iron chelation therapy can reduce inflammation and lesion volume following CNS injury, potentially via reductions in ROS release [25–27]. We suspect that iron chelation therapy protects microglia from excessive iron and prevents the interaction between free iron and activated microglia.

Both NOX2 and NOX4 inhibition in microglia previously exposed to FeSO_4_ and LPS significantly increased the presence of neurons in co-culture. This is consistent with recent findings that NOX2 and NOX4 mRNA silencing improved neuronal survivability by preventing apoptosis in a subarachnoid hemorrhage model [6]. In addition, multiple studies suggest improvement of outcomes with various NOX2, NOX4, and non-specific NOX inhibitors, which may be related to their contributions to reduced iron accentuated ROS production after TBI in vivo [44, 45].

Due to the heterogeneous nature of primary mixed neuronal cell cultures, we co-cultured microglia with differentiated PC12 neuronal-like cells to isolate the effect of iron accentuated ROS production on neurons. PC12s are neuron-like cells capable of neurite extension and therefore an ideal model to measure the severity of oxidative stress through the apoptotic marker cleaved caspase 3 [46]. Despite PC12’s reportedly robust nature, the cultures displayed a significantly increased count of cleaved caspase 3-positive cells after co-culture with microglia previously treated with FeSO_4_ & LPS & DMSO (Figs. 4 and 5). Inhibition of either NOX2 with GSK2795039 or NOX4 with GKT137831 in microglia prior to co-culture reduced the cellular toxicity among PC12s as evidenced by fewer apoptotic cells. This illustrates the cytotoxic potency of the ROS accentuation by FeSO_4_ & LPS and its indirect effect on a cell line reported to have unusually effective antioxidant capabilities.

## Conclusions

The data presented here provide evidence that microglia may passively and indirectly exacerbate neuronal toxicity after CNS damage. These data have been produced in both primary microglial cells and the microglial cell line BV2. To reduce animal use, experiments were primarily conducted in the cell line and confirmed with primary cell line experimentation where possible and most effective. Based on these data, the BV2 cell line appeared to be an acceptable cell for similar responses to primary microglia.

Iron has been identified as an important contributor to oxidative stress in neurodegenerative diseases and seems to be a significant player within TBI pathophysiology as well. Its focus as a primary contributor to oxidative stress is not novel; however, its mitigation in TBI may yield improved therapeutic outcomes by utilizing NOX2 or NOX4 inhibitors. Application of iron chelation or NOX inhibition to CNS injury seems promising [26, 42, 47]. Since effective treatments of CNS injury remain elusive, this may prove to be an effective strategy in the preservation of neurons and ultimately improved functional outcomes.

## Additional files

Additional file 1: **Figure S1.** BV2 cells express both DMT1 and light & heavy chain ferritin and take up iron. a) Western blotting of DMT1 revealed increased expression of those groups treated with LPS. b) Gene expression of light and heavy chain ferritin displayed a significant increase in light chain ferritin within FeSO_4_ groups. Heavy chain ferritin revealed a more robust response to LPS with groups exposed to LPS increasing gene expression. All groups were compared using two-way ANOVA with Tukey post-test. *N* = 4/group. *p < 0.05, **p < 0.01, ****p < 0.0001. Bars represent mean +/− SEM. c) Quantitation of BV2 microglia incorporation of iron from FeSO_4_ was done at 6 and 24 h post-iron exposure. FeSO_4_ at 10, 25, 50 or 100 μM resulted in a concentration dependent increase in iron incorporation that peaked at 100 μM. Addition of LPS led to a further increase at 24 h. *p < 0.05 vs control, **p < 0.01 vs control, two-way ANOVA with Dunnett’s multiple comparisons post-test. *N* = 2 technical replicates. (PDF 2265 kb)

Additional file 2: **Figure S2.** LDH assay for media from cells treated in the ROS assays in Fig. 1. No significant differences were noted among the treatment groups. X-axis represents FeSO_4_(a), Fe(NH_4_)_2_(SO_4_)_2_(b), NA_2_SO_4_(c). Within the DFO graph, the X-axis represents μM concentrations of FeSO_4_ (d). LDH release was also assessed in primary microglia with FeSO_4_ at 100 μM (e). All graphs represent an *n* = 5. All statistics are one-way ANOVA with Tukey post hoc test. Bars represent mean +/− SEM. (PDF 2065 kb)

Additional file 3: **Figure S3.** Immunocytochemistry for CD86 in primary microglia shows that iron does not exacerbate M1 polarization. Immunolabeling for CD86 (red) did not show a marked increase with addition of FeSO_4_. No detectable difference in cell number (as qualitatively assessed by DAPI (blue) staining) was noted between groups. Size bar = 50 μm. (TIF 2223 kb)

## Abbreviations

CNS: Central nervous system
DFO: Deferoxamine mesylate
DMT1: Divalent metal transporter 1
FACS: Fluorescence-activated cell sorting
FBS: Fetal bovine serum
FER: Ferritin
H_2_DCFDA: 2′,7′-Dichlorodihydrofluorescein diacetate
LPS: Lipopolysaccharide
NOX: NADPH oxidase
ROS: Reactive oxygen species
TBI: Traumatic brain injury
