# Supplementary figures and images for: Iron accentuated reactive oxygen species release by NADPH oxidase in activated microglia contributes to oxidative stress in vitro
Source: J Neuroinflammation. 2019 Feb 18;16:41. doi: 10.1186/s12974-019-1430-7 (PMC6378754; doi:10.1186/s12974-019-1430-7)

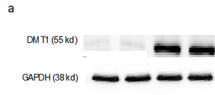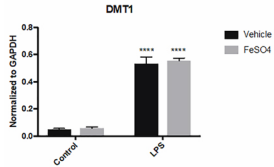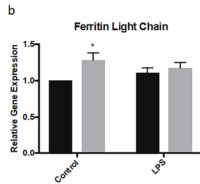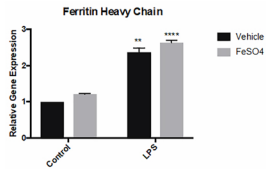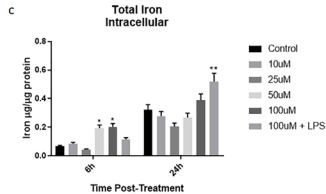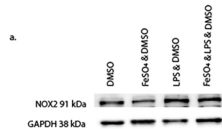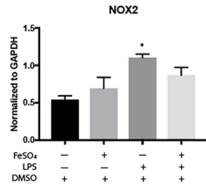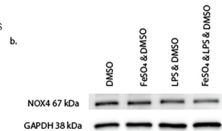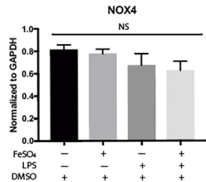

Supplement: Supplementary file 1 — Figure S1. BV2 cells express both DMT1 and light & heavy chain ferritin and take up iron. a) Western blotting of DMT1 revealed increased expression of those groups treated with LPS. b) Gene expression of light and heavy chain ferritin displayed a significant increase in light chain ferritin within FeSO4 groups. Heavy chain ferritin revealed a more robust response to LPS with groups exposed to LPS increasing gene expression. All groups were compared using two-way ANOVA with Tukey post-test. N = 4/group. *p < 0.05, **p < 0.01, ****p < 0.0001. Bars represent mean +/− SEM. c) Quantitation of BV2 microglia incorporation of iron from FeSO4 was done at 6 and 24 h post-iron exposure. FeSO4 at 10, 25, 50 or 100 μM resulted in a concentration dependent increase in iron incorporation that peaked at 100 μM. Addition of LPS led to a further increase at 24 h. *p < 0.05 vs control, **p < 0.01 vs control, two-way ANOVA with Dunnett’s multiple comparisons post-test. N = 2 technical replicates. (PDF 2265 kb) [file 12974_2019_1430_MOESM1_ESM.pdf]

$\text{FeSO}_4$ 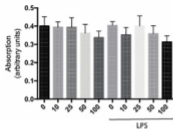 $\text{Fe}(\text{NH}_4)_2(\text{SO}_4)_2$ 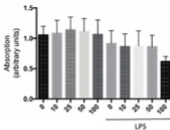 $\text{Na}_2\text{SO}_4$ 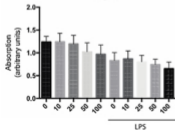

DFO

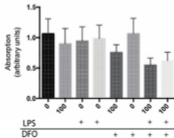 $\text{FeSO}_4$   
Primary Microglia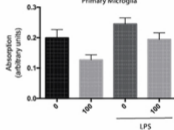

Supplement: Supplementary file 2 — Figure S2. LDH assay for media from cells treated in the ROS assays in Fig. 1. No significant differences were noted among the treatment groups. X-axis represents FeSO4(a), Fe(NH4)2(SO4)2(b), NA2SO4(c). Within the DFO graph, the X-axis represents μM concentrations of FeSO4 (d). LDH release was also assessed in primary microglia with FeSO4 at 100 μM (e). All graphs represent an n = 5. All statistics are one-way ANOVA with Tukey post hoc test. Bars represent mean +/− SEM. (PDF 2065 kb) [file 12974_2019_1430_MOESM2_ESM.pdf]

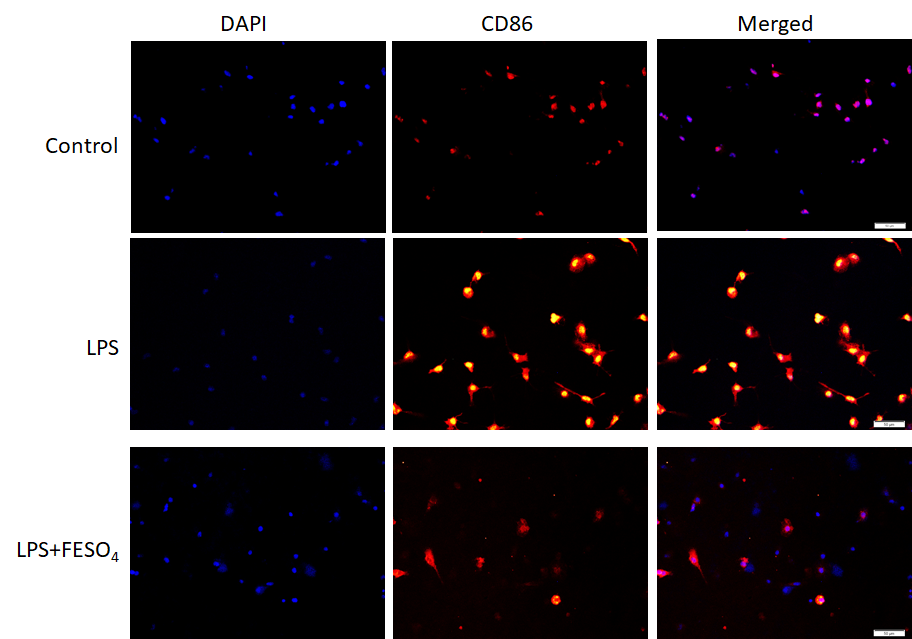

Supplement: Supplementary file 3 — Figure S3. Immunocytochemistry for CD86 in primary microglia shows that iron does not exacerbate M1 polarization. Immunolabeling for CD86 (red) did not show a marked increase with addition of FeSO4. No detectable difference in cell number (as qualitatively assessed by DAPI (blue) staining) was noted between groups. Size bar = 50 μm. (TIF 2223 kb) [file 12974_2019_1430_MOESM3_ESM.tif]
